# Association of *TLR3* L412F Polymorphism with Cytomegalovirus Infection in Children

**DOI:** 10.1371/journal.pone.0169420

**Published:** 2017-01-03

**Authors:** Mirosława Studzińska, Agnieszka Jabłońska, Małgorzata Wiśniewska-Ligier, Dorota Nowakowska, Zuzanna Gaj, Zbigniew J. Leśnikowski, Teresa Woźniakowska-Gęsicka, Jan Wilczyński, Edyta Paradowska

**Affiliations:** 1 Laboratory of Molecular Virology and Biological Chemistry, Institute of Medical Biology, Polish Academy of Sciences, Lodz, Poland; 2 Department of Pediatrics, Immunology, and Nephrology, Polish Mother's Memorial Hospital Research Institute, Lodz, Poland; 3 Department of Perinatology and Gynecology, Polish Mother’s Memorial Hospital Research Institute, Lodz, Poland; 4 3rd Department of Pediatrics, Polish Mother’s Memorial Hospital Research Institute, Lodz, Poland; 5 2nd Department of Obstetrics and Gynecology, Warsaw Medical University, Warsaw, Poland; University of St Andrews, UNITED KINGDOM

## Abstract

Intracellular Toll-like receptor 3 (TLR3) recognizes viral double-stranded RNA (dsRNA) and activates antiviral immune responses through the production of type I interferons (IFNs) and inflammatory cytokines. This receptor binds to dsRNA molecules produced during human cytomegalovirus (HCMV) replication. TLR7 senses viral single-stranded RNA (ssRNA) in endosomes, and it can interact with endogenous RNAs. We determined the genotype distribution of single-nucleotide polymorphisms (SNPs) within the *TLR3* and *TLR7* genes in children with HCMV infection and the relationship between *TLR* polymorphisms and viral infection. We genotyped 59 children with symptomatic HCMV infection and 78 healthy individuals for SNPs in the *TLR3* (rs3775290, c.1377C>T, F459F; rs3775291, c.1234C>T, L412F; rs3775296, c.-7C>A) and *TLR7* (rs179008, c.32A>T, Q11L; rs5741880, c.3+1716G>T) genes. SNP genotyping was performed using polymerase chain reaction-restriction fragment length polymorphism (PCR-RFLP) and capillary electrophoresis. The HCMV DNA load was quantified by real-time PCR. We found an increased frequency of the heterozygous genotype *TLR3* L412F in children with HCMV infection compared with uninfected cases. In individuals with a mutation present in at least one allele of the L412F SNP, an increased risk of HCMV disease was found, and this result remained highly significant after Bonferroni’s correction for multiple testing (*P*^*c*^ < 0.001). The heterozygous genotype of this SNP was associated with the increased risk of HCMV disease in an adjusted model that included the HCMV DNA copy number in whole blood and urine (*P* < 0.001 and *P* = 0.008, respectively). Moreover, those with a heterozygous genotype of rs3775296 showed an increased relative risk of HCMV infection (*P* = 0.042), but this association did not reach statistical significance after correction for multiple testing. In contrast, the rs3775290 SNP of *TLR3* and *TLR7* SNPs were not related to viral infection. A moderate linkage disequilibrium (LD) was observed between the SNPs rs3775291 and rs3775296 (r^2^ = 0.514). We suggest that the L412F polymorphism in the *TLR3* gene could be a genetic risk factor for the development of HCMV disease.

## Introduction

Human cytomegalovirus (HCMV) is a widespread opportunistic ß-herpesvirus that has latently infected approximately 60–100% of the world’s population [1]. It is also the most common cause of congenital viral infection in the developed world, occurring in 0.5–2.0% of pregnancies [2, 3]. HCMV infection is usually asymptomatic, although the virus remains latent throughout life and may reactivate at any time. Among immunosuppressed patients and newborns with congenital infections, the virus provokes a number of disparate outcomes. A number of factors can affect the interaction between the host immune system and the virus, and the major determinants of HCMV disease appear to be host factors.

Host-viral interactions are initiated *via* host recognition of pathogen-associated molecular patterns (PAMPs) of the virus. This recognition occurs through host pattern recognition receptors (PRRs), including Toll-like receptors (TLRs), RIG-I-like receptors (RLRs), NOD-like receptors (NLRs), and C-type lectin receptors (CLRs). Human TLRs are present on the cell membrane (TLR1, -2, -4, -5, -6) and within endosomal compartments (TLR3, -7, -8, and -9), while NLRs and RIG-I helicases are intracellular sensors. Viral RNA is recognized by endosomal TLRs, such as TLR3, -7, and -8, as well as by TLR-independent pathways, and can activate the cytoplasmic RNA helicases RIG-I and MDA5 [4–7]. TLR3 recognizes poly(I:C), a synthetic double-stranded RNA (dsRNA) analog, as well as viral dsRNA that can be generated as an intermediate during the replication cycle of single-stranded RNA (ssRNA) or DNA viruses [8–10]. Interactions of intracellular TLR3 with murine CMV (MCMV) are related to the binding of the dsRNA molecules produced during virus replication [11]. TLR7 and TLR8 detect viral and non-viral ssRNA [12, 13], while TLR9 recognizes unmethylated cytosine-phosphate-guanosine (CpG) motifs from DNA viruses, including MCMV and herpes simplex virus (HSV-1/HSV-2) [14, 15].

Few studies have suggested a role of *TLR* polymorphisms in the HCMV-associated disease pathology. Associations between *TLR2* [16, 17], *TLR4* [18], and *TLR9* polymorphisms [19–22] with HCMV infection have been found. It was previously demonstrated that the *TLR9*–1486 and 2848 polymorphisms as well as the *TLR2* 1350 SNP are associated with an enhanced risk of HCMV infection and disease in newborns and infants [23–25]. However, little is known about the role of *TLR3* and *TLR7* polymorphisms in the pathogenesis of cytomegaly. *In vitro* experiments revealed that fibroblasts isolated from carriers of the L412F variant of *TLR3* showed reduced IFNγ and TNFα secretion in response to HCMV stimulation [26]. *TLR7* polymorphisms have been suggested to play a role in the development of glycoprotein B (gB) antibodies following HCMV immunization. Women that were homozygous carriers of four *TLR7* SNPs (rs179008, rs179009, rs179013 and rs179018) demonstrated a higher vaccination-induced antibody response to HCMV gB than did heterozygotes or homozygotes for the common allele [27].

The objective of the current study is to determine the frequencies of SNPs in the *TLR3* (rs3775290, rs3775291, rs3775296) and *TLR7* (rs179008, and rs5741880) genes and investigate the associations between these polymorphisms and HCMV infection in children. The majority of the children in this study were also involved in earlier studies on the relationship between polymorphisms in the *TLR2*, *TLR4*, and *TLR9* genes and HCMV infection [24, 28]. In an attempt to provide more definitive conclusion, all previously tested *TLR* SNPs: *TLR2* (rs121917864 and rs5743708), *TLR4* (rs4986790), and *TLR9* (rs5743836, rs187084, rs352139, and rs352140) were included in multiple testing analysis.

## Materials and Methods

### Patients

From February 2008 to January 2011, 59 children with HCMV infection (median age 4 months; range 1–24 months) were selected for the study at the 3rd Department of Pediatrics of the Polish Mother’s Memorial Hospital Research Institute in Lodz, Poland. Fifty-nine children with HCMV infection and some uninfected infants were earlier examined for the presence of *TLR2*, *TLR4*, and *TLR9* SNPs as described in our previous studies [24, 28]. Among uninfected infants, 30 out of 78 cases were analyzed for the presence of *TLR2* and *TLR4* SNPs [28], while 42 out of 78 were genotyped for *TLR9* SNPs. HCMV infection was confirmed by the presence of HCMV-specific antibodies and/or HCMV DNA detection in whole blood and/or urine samples. As previously described [24], serum samples were assessed for anti-HCMV IgG and IgM antibodies. The whole blood and urine samples were collected during the exacerbation of cytomegaly symptoms, including anemia, thrombocytopenia, pneumonia, hepatitis, hepatic dysfunction, and others. Due to selection bias, all children with HCMV infection in this study were symptomatic. Because the samples for virus detection were collected after 3 weeks of life, all children were classified as having postnatal or unproven congenital HCMV infection. The demographic and clinical characteristics of patients with HCMV infection were summarized in Table 1. HCMV-positive children were compared with 78 unrelated seronegative and aviremic newborn infants. All subjects were of European descent, and there were no ethnic differences between the cases and the uninfected group.

**Table 1:** Demographic and clinical characteristics of study subjects with HCMV infection.

Characteristics	
**Total No.**	59
**Mean ± SD age, months**	5.7 ± 5.3
**Median (range), months**	4 (1–24)
**Gender number; n (%)**^a^	
Female	20 (33.9)
Male	39 (66.1)
**Symptoms; n (%)**^a^	
Hematological disorders	25 (42.4)
Pneumonia	16 (27.1)
Liver damage or hepatitis	15 (25.4)
Neurological dysfunction	15 (25.4)
CNS damage	14 (23.7)
Psychomotor retardation	12 (20.3)
Jaundice	11 (18.6)
Hearing loss	6 (10.2)
IUGR	5 (8.5)
Hepatosplenomegaly	5 (8.5)
Heart disease	2 (3.4)
**Anti-HCMV serologic status**	
IgG-positive, IgM-negative	25 (42.4)
IgG-positive, IgM-positive	32 (54.2)
IgG-negative, IgM-negative	2 (3.4)

^a^Values are the number of infants (%).

The study protocols were approved by the Bioethics Committee of the Medical University of Lodz (RNN/120/09/KE) and the Ethics Committee of the Polish Mother’s Memorial Hospital Research Institute. Parents or guardians provided written informed consent on behalf of the children to participate in this study.

### *TLR* SNP analysis

**Fig 1:**
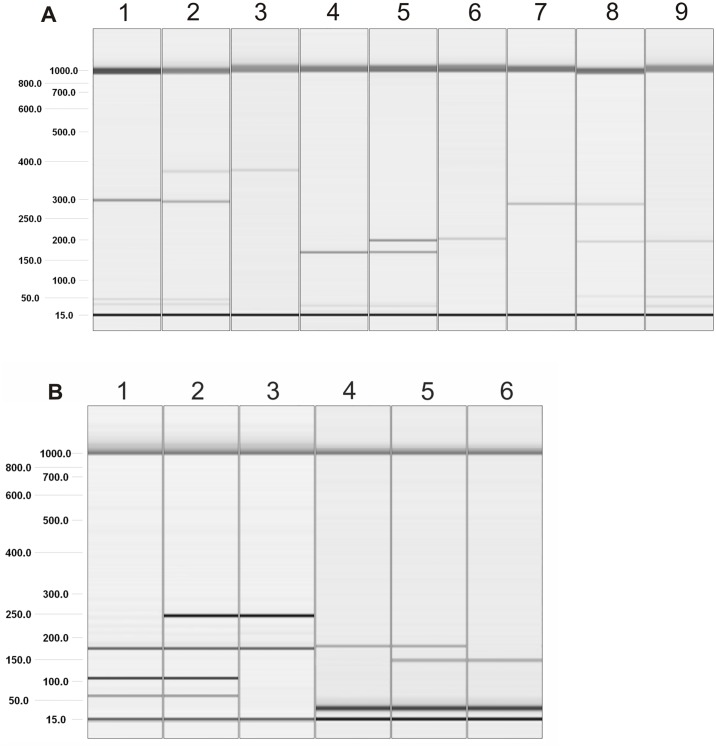
Visualization of PCR-RFLP products for *TLR3* SNP (A) and *TLR7* SNP (B) genotyping. Gel image: A) rs3775290: 1. wild type CC, 2. heterozygous CT, 3. homozygous recessive TT; rs3775291: 4. wild type CC, 5. heterozygous CT, 6. homozygous recessive TT; rs3775296: 7. wild type CC, 8. heterozygous CA, 9. homozygous recessive AA. B) rs179008: 1. wild type AA, 2. heterozygous AT, 3. homozygous recessive TT; rs5741880: 4. wild type GG, 5. heterozygous GT, 6. homozygous recessive TT. Alignment markers (15 bp, 1 kbp).

Genomic DNA was extracted from EDTA-anticoagulated peripheral blood using the QIAamp DNA Blood Mini Kit (Qiagen, Hilden, Germany) according to the manufacturer’s recommendations. The concentration and purity of DNA were measured using a NanoDrop 2000c UV-vis Spectrophotometer (Thermo Scientific, Wilmington, DE, USA). A total of 5 SNPs in the *TLR3* (rs3775290, rs3775291, and rs3775296) and *TLR7* (rs179008, rs5741880) genes were analyzed (Table 2). The selection of SNPs was based on their possible functional effect and due to the existence of previous associations with infectious diseases. The identification of SNPs was performed by polymerase chain reaction-restriction fragment length polymorphism (PCR-RFLP) using primers described previously by Noguchi et al. [29] (*TLR3* SNPs), Cheng et al. [30] (*TLR7* SNP rs179008), and Arslan et al. [31] (*TLR7* SNP rs5741880). Each PCR reaction contained 200 ng of genomic DNA, primers (1 μM each), dNTPs (2.5 mM), 10x *Taq* buffer (100 mM Tris-HCl, 500 mM KCl, 0.8% Nonidet-P40; pH 8.8), 2 mM MgCl_2_, and 1 U of *Taq* DNA Polymerase (Fermentas, Glen Burnie, MD, USA). The thermal cycling conditions for *TLR3* were 5 min at 95°C and 35 cycles each of 45 s at 95°C, 15 s at 56°C, and 30 s at 72°C. The PCR parameters for *TLR7* were as follows: 5 min at 94°C and 35 cycles each of 30 s at 94°C, 30 s at 52°C, and 1 min. at 72°C. The final extension was at 72°C for 7 min. The reactions were performed in the Veriti thermal cycler (Applied Biosystems, Foster City, CA, USA). The PCR-amplified fragments corresponding to the *TLR3* rs3775290 (c.1377C>T, F459F), rs3775291 (c.1234C>T, L412F), rs3775296 (c.-7C>A) and *TLR7* rs179008 (c.32A>T, Q11L), and rs5741880 (c.3+1716G>T) polymorphisms were digested with the restriction enzymes *Taq*I, *HpyF3*I, *Mbo*II, *Xap*I, and *Hpy*188I, respectively (Fermentas, Hanover, MD, USA). The digested fragments were separated using the QIAxcel DNA Screening Kit (Qiagen) on the QIAxcel capillary electrophoresis system (Qiagen). The retention time of the PCR fragments relative to the QX Alignment Marker 15 bp/1 kb fragments was calculated using the BioCalculator software (Qiagen). The PCR-RFLP product sizes were determined by comparing the retention time with the QX DNA Size Marker 50–800 bp. The *TLR3* and *TLR7* SNPs were identified by different fragment lengths described in Table 3 and presented in Fig 1. For two SNPs, rs3775290 (allele C) and rs3775296 (allele A), the presence of three instead of two products was observed in some samples, possibly due to degraded DNA. We randomly selected 20% of the samples to validate the results of genotyping by PCR-RFLP assay. The obtained results were validated by direct sequencing of selected PCR products using the MiSeq system (Illumina, San Diego, CA, USA). *TLR2*, *TLR4*, and *TLR9* genotyping was done using PCR-RFLP method. Details of primers, PCR conditions, and restriction enzymes have been described elsewhere [24, 28]. Digested PCR products were analyzed using the QIAxcel capillary electrophoresis system (Qiagen).

**Table 2:** Selected single-nucleotide polymorphisms in the *TLR3* and *TLR7* genes.

Gene	SNP ID	Alleles	Amino acid change	Function	MAF		
					Polish	European	Global
***TLR3***	rs3775290	C/T	F459F	Silent	0.321	0.273	0.272
	rs3775291	C/T	L412F	Missense	0.256	0.324	0.232
	rs3775296	C/A		UTR-5	0.173	0.174	0.182
***TLR7***	rs179008	A/T	Q11L	Missense	0.301	0.176	0.118
	rs5741880	G/T		Intronic	0.308	0.092	0.207

MAF, Minor allele frequency in the Polish population (present study), European and Global population (NCBI, 1000 Genomes Project)

**Table 3:** Restriction enzymes and length of the restriction fragments.

Gene	SNP	Restriction enzyme	Genotype	Length of the restriction fragments (bp)
***TLR3***	rs3775290	*Taq*I	CC	39, 45, 299
			CT	39, 45, 299, 383
			TT	383
	rs3775291	*HpyF3I*	CC	31,169
			CT	31, 169, 200
			TT	200
	rs3775296	*Mbo*II	CC	285
			CA	37, 52, 196, 285
			AA	37, 52, 196
***TLR7***	rs179008	*XapI*	AA	64, 116, 174
			AT	64, 116, 174, 247
			TT	174, 247
	rs5741880	*Hpy*188I	GG	186
			GT	43, 143, 186
			TT	43, 143

bp, base pairs

### Quantification of HCMV DNA

The HCMV DNA copy number in DNA isolates from whole blood and urine samples were determined using a real-time PCR assay targeting the *UL55* gene using a 7900HT Fast Real-Time PCR System (Applied Biosystems), as described elsewhere [24, 32]. The lower limit of detection of this assay was 100 HCMV DNA copies/mL.

### Statistical analysis

*TLRs* genotype and allele frequencies were calculated by direct counting. All the statistical analyses, including the Hardy-Weinberg equilibrium (HWE) test and the associations of genotypes and haplotypes with HCMV infection status, were performed using the SNPSTATS program (http://bioinfo.iconcologia.net/index.php?module=Snpstats) [33]. Odds ratio (OR) and 95% confidence intervals (95% CIs) with both unadjusted and adjusted multivariate models were calculated to examine the association between the *TLR* SNPs and HCMV infection. To adjust for possible confounders, the linear model was extended to include the effects of viral load, which is a key risk factor for the development of HCMV disease. Linkage disequilibrium (LD) was analyzed by Haploview software (version 4.2; Broad Institute, Cambridge, MA, USA; http://www.broad.mit.edu/mpg/haploview) [34]. The significance of differences in the estimated allele frequencies between HCMV-infected and uninfected patient groups were examined on Haploview 4.2 using χ2 tests, and alleles were corrected using permutation test after running 1000 times. The association between the *TLR* genotype and the symptoms based on logistic regression were performed using the SPSS statistical software package for Windows 17.0 (SPSS, Chicago, IL, USA). The distribution of alleles and genotypes among the study groups was examined using the Fisher’s exact test. The one-way analysis of variance (ANOVA) and Mann-Whitney U tests were used to study the association between *TLR* polymorphisms and viral load. For all statistical tests a *P*-value of ≤ 0.05 was considered to be statistically significant. Bonferroni’s correction (*P*^*c*^) of the significance level was applied to account for multiple testing. The *P*^*c*^-values were count by dividing the *P*-value by the number of comparisons.

## Results

### Study population

Viral DNA was detected and quantified in 49 of the 59 examined children with HCMV infection (83%) by real-time PCR. Among them, HCMV DNA was detected in 37/49 whole blood (75.5%) and 42/49 urine (85.7%) samples. In the remaining 10 cases, active HCMV infection was confirmed by the detection of HCMV-specific IgM antibodies. Cytomegalovirus serology assays were performed for all 59 HCMV-infected children and 78 control newborn infants. Sera from 32 infected children were positive for IgG and IgM, 25 were positive for IgG only, and 2 were IgG- and IgM-negative (see Table 1). The anti-HCMV IgG and IgM serology results were negative for all control infants.

### Frequency of *TLR3* and *TLR7* gene polymorphisms in children

To analyze the impact of *TLR3* and *TLR7* SNPs on the incidence of HCMV infection, a group of uninfected and HCMV-infected children was analyzed with different genetic models. To explore the data, we performed the analysis of genotype and allele frequencies of *TLR* polymorphisms, including *TLR2*, *TLR4*, and *TLR9* SNPs in the patient groups examined (see Tables 4 and 5). Fifty-nine cases of HCMV infection and part of uninfected subjects has been described previously in detail for *TLR2*, *TLR4* [28], and *TLR9* SNPs [24]. The genotype and allele frequencies for all studied *TLR2*, *TLR3*, *TLR7*, and *TLR9* SNPs were in HWE in the uninfected group, while *TLR4* SNP was not (*P* ≤ 0.050) and was excluded from further analysis. With the exception of the rs3775291 SNP in the *TLR3* gene and the rs5743708 SNP in the *TLR9* gene, all polymorphisms were also in HWE in HCMV cases.

**Table 4:** Frequency and distribution of *TLR* genotypes and their association with HCMV infection.

Gene	SNP	Model	Genotype	Genotype frequencies; n (%)^a^		Unadjusted		Adjusted^b^	
				HCMV-infected	Uninfected	OR (95% CI)	*P*^c^	OR (95% CI)	*P*^*c*^
***TLR2***	rs121917864	Codominant	CC	59 (100%)	77 (98.7%)	1.00	0.29	1.00	0.48
			CT	0 (0%)	1 (1.3%)	0.00 (0.00-NA)		0.00 (0.00-NA)	
	rs5743708	Codominant	GG	57 (96.6%)	78 (100%)	1.00	0.065	1.00	NA
			GA	2 (3.4%)	0 (0%)	NA (0.00-NA)		NA (0.00-NA)	
***TLR3***	rs3775290	Codominant	CC	24 (40.7)	38 (48.7)	1.00	0.33	1.00	0.042
			CT	30 (50.9)	30 (38.5)	1.58 (0.77–3.25)		3.17 (1.10–9.15)	
			TT	5 (8.5)	10 (12.8)	0.79 (0.24–2.60)		0.63 (0.07–5.88)	
		Dominant	CC	24 (40.7)	38 (48.7)	1.00	0.35	1.00	0.068
			CT-TT	35 (59.3)	40 (51.3)	1. 39 (0.70–2.74)		2.53 (0.90–7.15)	
		Recessive	CC-CT	54 (91.5)	68 (87.2)	1.00	0.41	1.00	0.23
			TT	5 (8.5)	10 (12.8)	0.63 (0.20–1.95)		0.32 (0.04–2.68)	
		Overdominant	CC-TT	29 (49.1)	48 (61.5)	1.00	0.15	1.00	0.013
			CT	30 (50.9)	30 (38.5)	1.66 (0.83–3.28)		3.43 (1.25–9.38)	
	rs3775291	Codominant	CC	5 (8.5)	46 (59.0)	1.00	**<0.0001**	1.00	**0.0001**
			CT	49 (83.0)	24 (30.8)	18.78 (6.61–53.36)		11.50 (3.08–42.97)	
			TT	5 (8.5)	8 (10.2)	5.75 (1.35–24.49)		1.92 (0.18–20.80)	
		Dominant	CC	5 (8.5)	46 (59.0)	1.00	**<0.0001**	1.00	**0.0001**
			CT-TT	54 (91.5)	32 (41.0)	15.52 (5.59–43.11)		9.10 (2.48–33.36)	
		Recessive	CC-CT	54 (91.5)	70 (89.7)	1.00	0.72	1.00	0.38
			TT	5 (8.5)	8 (10.3)	0.81 (0.25–2.62)		0.42 (0.05–3.52)	
		Overdominant	CC-TT	10 (16.9)	54 (69.2)	1.00	**<0.0001**	1.00	**<0.0001**
			CT	49 (83.1)	24 (30.8)	11.02 (4.79–25.36)		10.13 (3.09–33.13)	
	rs3775296	Codominant	CC	32 (54.2)	55 (70.5)	1.00	0.12	64	0.55
			CA	24 (40.7)	19 (24.4)	2.17 (1.03–4.56)		1.78 (0.64–4.96)	
			AA	3 (5.1)	4 (5.1)	1.29 (0.27–6.13)		1.06 (0.11–10.27)	
		Dominant	CC	32 (54.2)	55 (70.5)	1.00	0.05	1.00	0.32
			CA-AA	27 (45.8)	23 (29.5)	2.02 (1.00–4.09)		1.66 (0.62–4.41)	
		Recessive	CC-CA	56 (94.9)	74 (94.9)	1.00	0.99	1.00	0.91
			AA	3 (5.1)	4 (5.1)	0.99 (0.21–4.61)		0.88 (0.09–8.31)	
		Overdominant	CC-AA	35 (59.3)	59 (75.6)	1.00	0.042	1.00	0.27
			CA	24 (40.7)	19 (24.4)	2.13 (1.02–4.43)		1.77 (0.65–4.88)	
***TLR7***	rs179008	Codominant	AA	35 (59.3)	39 (50.0)	1.00	0.51	1.00	0.5
			AT	18 (30.5)	31 (39.7)	0.65 (0.31–1.35)		0.54 (0.19–1.57)	
			TT	6 (10.2)	8 (10.3)	0.84 (0.26–2.65)		0.70 (0.13–3.68)	
		Dominant	AA	35 (59.3)	39 (50.0)	1.00	0.28	1.00	0.25
			AT-TT	24 (40.7)	39 (50.0)	0.69 (0.35–1.36)		0.57 (0.22–1.52)	
		Recessive	AA-AT	53 (89.8)	70 (89.7)	1.00	0.99	1.00	0.87
			TT	6 (10.2)	8 (10.3)	0.99 (0.32–3.03)		0.87 (0.17–4.45)	
		Overdominant	AA-TT	41 (69.5)	47 (60.3)	1.00	0.26	1.00	0.28
			AT	18 (30.5)	31 (39.7)	0.67 (0.33–1.36)		0.57 (0.20–1.61)	
	rs5741880	Codominant	GG	24 (40.7)	37 (47.4)	1.00	0.57	1.00	0.17
			GT	31 (52.5)	34 (43.6)	1.41 (0.69–2.85)		0.36 (0.12–1.11)	
			TT	4 (6.8)	7 (9.0)	0.88 (0.23–3.34)		0.70 (0.13–3.79)	
		Dominant	GG	24 (40.7)	37 (47.4)	1.00	0.43	1.00	0.082
			GT-TT	35 (59.3)	41 (52.6)	1.32 (0.66–2.61)		0.42 (0.15–1.15)	
		Recessive	GG-GT	55 (93.2)	71 (91.0)	1.00	0.64	1.00	0.99
			TT	4 (6.8)	7 (9.0)	0.74 (0.21–2.65)		1.01 (0.20–5.27)	
		Overdominant	GG-TT	28 (47.5)	44 (56.4)	1.00	0.3	1.00	0.068
			GT	31 (52.5)	34 (43.6)	1.43 (0.73–2.83)		0.38 (0.13–1.14)	
***TLR9***	rs5743836	Codominant	TT	58 (98.3%)	78 (100%)	1.00	0.19	1.00	0.08
			CC	1 (1.7%)	0 (0%)	NA (0.00-NA)		NA (0.00-NA)	
	rs187084	Codominant	TT	17 (28.8%)	31 (39.7%)	1.00	0.085	1.00	0.096
			TC	36 (61%)	33 (42.3%)	1.99 (0.93–4.24)		2.82 (0.92–8.68)	
			CC	6 (10.2%)	14 (17.9%)	0.78 (0.25–2.41)		0.89 (0.15–5.13)	
		Dominant	TT	17 (28.8%)	31 (39.7%)	1.00	0.18	1.00	0.13
			TC-CC	42 (71.2%)	47 (60.3%)	1.63 (0.79–3.36)		2.24 (0.75–6.71)	
		Recessive	TT-TC	53 (89.8%)	64 (82%)	1.00	0.19	1.00	0.29
			CC	6 (10.2%)	14 (17.9%)	0.52 (0.19–1.44)		0.46 (0.10–2.19)	
		Overdominant	TT-CC	23 (39%)	45 (57.7%)	1.00	0.03	1.00	0.031
			TC	36 (61%)	33 (42.3%)	2.13 (1.07–4.25)		2.92 (1.07–7.97)	
	rs352139	Codominant	AA	33 (55.9%)	25 (32%)	1.00	**0.0011**	1.00	0.035
			GA	23 (39%)	34 (43.6%)	0.51 (0.24–1.08)		0.55 (0.20–1.51)	
			GG	3 (5.1%)	19 (24.4%)	0.12 (0.03–0.45)		0.11 (0.01–0.92)	
		Dominant	AA	33 (55.9%)	25 (32%)	1.00	0.005	1.00	0.057
			GA-GG	26 (44.1%)	53 (68%)	0.37 (0.18–0.75)		0.39 (0.15–1.03)	
		Recessive	AA-GA	56 (94.9%)	59 (75.6%)	1.00	**0.0013**	1.00	0.021
			GG	3 (5.1%)	19 (24.4%)	0.17 (0.05–0.59)		0.15 (0.02–1.17)	
		Overdominant	AA-GG	36 (61%)	44 (56.4%)	1.00	0.59	1.00	0.82
			GA	23 (39%)	34 (43.6%)	0.83 (0.42–1.65)		0.90 (0.34–2.34)	
	rs352140	Codominant	CC	17 (28.8%)	44 (56.4%)	1.00	0.0051	1.00	0.017
			CT	32 (54.2%)	26 (33.3%)	3.19 (1.49–6.83)		4.40 (1.41–13.75)	
			TT	10 (16.9%)	8 (10.3%)	3.24 (1.09–9.58)		4.40 (0.97–20.02)	
		Dominant	CC	17 (28.8%)	44 (56.4%)	1.00	**0.0011**	1.00	**0.0043**
			CT-TT	42 (71.2%)	34 (43.6%)	3.20 (1.56–6.57)		4.40 (1.47–13.13)	
		Recessive	CC-CT	49 (83%)	70 (89.7%)	1.00	0.25	1.00	0.33
			TT	10 (16.9%)	8 (10.3%)	1.79 (0.66–4.85)		1.94 (0.53–7.19)	
		Overdominant	CC-TT	27 (45.8%)	52 (66.7%)	1.00	0.014	1.00	0.03
			CT	32 (54.2%)	26 (33.3%)	2.37 (1.18–4.75)		2.89 (1.09–7.63)	

^a^Values are the number of examined infants (%);

^b^Adjusted analysis was carried out for HCMV DNA copy number in whole-blood samples;

OR: odds ratio; 95% CI: 95% confidence interval; *P*, logistic regression model; NA: not available; *P*^c^, The significance level after Bonferroni’s correction for multiple testing is 0.0045 (raw *P*-value/11).

**Table 5:** The distribution of allele frequencies of TLR SNPs in infants with and without HCMV infection.

Gene	SNP	Allele	Allele frequencies; n (%)^a^		*P*	*P*^*b*^
			HCMV-infected	Uninfected		
***TLR2***	rs121917864	C	118 (100)	155 (99.4)	0.3836	1.0000
		T	0 (0)	1 (0.6)		
	rs5743708	G	116 (98.3)	156 (100)	0.1027	0.8160
		A	2 (1.7)	0 (0)		
***TLR3***	rs3775290	C	78 (66.1)	106 (67.9)	0.7472	1.0000
		T	40 (33.9)	50 (32.1)		
	rs3775291	C	59 (50.0)	116 (74.4)	**<0.0001**	**0.0010**
		T	59 (50.0)	40 (25.6)		
	rs3775296	C	88 (74.6)	129 (82.7)	0.1012	0.6140
		A	30 (25.4)	27 (17.3)		
***TLR7***	rs179008	A	88 (74.6)	109 (69.9)	0.391	1.0000
		T	30 (25.4)	47 (30.1)		
	rs5741880	G	79 (67.0)	108 (69.2)	0.6879	1.0000
		T	39 (33.0)	48 (30.8)		
***TLR9***	rs5743836	T	116 (98.3)	156 (100)	0.1027	0.8160
		C	2 (1.7)	0 (0)		
	rs187084	T	70 (59.3)	95 (60.9)	0.7919	1.0000
		C	48 (40.7)	61 (39.1)		
	rs352139	G	29 (24.6)	72 (46.2)	**0.0002**	**0.0030**
		A	89 (75.4)	84 (53.8)		
	rs352140	C	66 (55.9)	114 (73.1)	**0.0031**	**0.0300**
		T	52 (44.1)	42 (26.9)		

^a^Values are the number of alleles (%); *P*, *P*-value was calculated from chi-square test; *P*^*b*^, *P*-value for estimated alleles were generated from 1000 permutations using the expectation-maximization (EM) algorithm.

The distribution of the rs3775291 and rs3775296 genotypes of the *TLR3* gene was different between HCMV-infected and uninfected individuals (see Table 4; *P* < 0.001 and *P* = 0.073, respectively; Fisher’s exact test). The frequency of the wild-type genotype of the *TLR3* SNP rs3775291 was significantly higher in uninfected than in HCMV-infected cases (59.0% vs. 8.5%; *P* < 0.0001; Fisher’s exact test). It should be noted that the heterozygous variant of this SNP was detected more frequently among children with HCMV infection than in uninfected infants (83.0% vs. 30.8%; *P*< 0.0001; Fisher’s exact test). Consequently, the wild-type rs3775291 C allele was detected more frequently in uninfected children (74.4%) compared with HCMV-infected cases (50.0%; *P* < 0.001; see Table 5). No difference was observed in the distribution of the *TLR3* SNP rs3775290 or in both *TLR7* SNPs between infected and uninfected children (*P* > 0.05). In the current study, analysis of the allele frequencies of *TLR9* SNPs showed that A (rs352139) and T (rs352140) alleles were detected more frequently in children with HCMV infection than in uninfected individuals (permutation values *P* = 0.003 and *P* = 0.03, respectively; Table 5). No differences in the frequency of *TLR2* and *TLR4* alleles were observed.

### *TLR3* L412F polymorphism is associated with the risk of HCMV infection

Single-SNP analysis revealed that a mutation present in at least one allele of the *TLR3* SNP rs3775291 was significantly associated with the risk of HCMV infection (see Table 4). Heterozygous CT and homozygous recessive TT genotypes at the 1234 locus were associated with a significantly increased risk of infection in almost all genetic models (OR 18.78, 95% CI 6.61–53.36 in the codominant model and OR 15.52, 95% CI 5.59–43.11 in the dominant model; *P* < 0.001). Moreover, the results suggested that the heterozygous CT genotype was mainly responsible for the observed association of this SNP with HCMV cases (OR 11.02, 95% CI 4.79–25.36; *P* < 0.001 in the overdominant model). These associations persisted even after adjustment for multiple comparisons using Bonferroni’s correction (*P*^*c*^ < 0.001). In addition, the heterozygous genotype of the rs3775296 SNP was associated with a 2-fold increased risk of HCMV infection (OR 2.13, 95% CI 1.02–4.43; *P* = 0.042 in the overdominant model). However, this association did not reach statistical significance after Bonferroni’s correction for multiple testing. No association between *TLR3* rs3775290 or any *TLR7* SNP genotypes and the risk of HCMV infection was found. In the examined children, an association of the *TLR9* SNPs rs352139 and rs352140 with HCMV infection has been confirmed (Table 5).

There was no significant association between the *TLR3* and *TLR7* SNPs and the presence of specific HCMV symptoms, excluding hepatitis. Analysis of genetic polymorphisms of the *TLRs* in the HCMV-infected children showed that hepatitis seemed to be correlated with the presence of some genotypes, although this symptom was observed in only a small number of examined children (5 cases). The TT genotype of *TLR3* rs3775290 was detected in 2/5 children with hepatitis and was associated with a 11-fold increased risk of hepatitis in the unadjusted and adjusted models (OR 11.3, 95% CI 1.34–95.82; *P* = 0.026), whereas the TT variant of *TLR7* rs179008 was associated with an 8-fold higher risk of this symptom (OR 8.33, 95% CI 1.06–65.30; *P* = 0.044). No significant association between the examined SNPs and other symptoms was detected.

### Associations between *TLR3* and *TLR7* SNPs and HCMV load

The viremia levels in HCMV-infected infants ranged from 0 to 3.57 × 10^5^ copies/mL (mean 7.54 × 10^3^ ± 4.64 × 10^4^ copies/mL) in the peripheral blood. The HCMV DNA load in urine samples ranged from 0 to 2.21 × 10^8^ copies/mL (mean 6.82 × 10^6^ ± 3.45 × 10^7^ copies/mL). Despite the considerable differences in viral load, we observed a correlation of the *TLR3* and *TLR7* SNPs with the HCMV DNA concentration in the bodily fluids. The children with heterozygous genotype of rs3775291 had approximately 10-fold increased risk of HCMV disease in an adjusted model that included the HCMV DNA copy number in the whole blood (OR 11.50, 95% CI 3.08–42.97, *P* < 0.001 in codominant model; Table 4). Similarly, an at least 5-fold increased risk of HCMV infection was observed in these children in an adjusted model that included the HCMV DNA load in urine samples (OR 7.03, 95% CI 1.79–27.62, *P* = 0.008 in codominant model; data not presented). This association reached statistical significance after Bonferroni’s correction in almost all genetic models (*P*^*c*^ < 0.01). The higher viremia levels were also detected in carriers of the heterozygous genotype of the *TLR7* SNP rs5741880 (*P* = 0.007; Mann-Whitney test). However, further studies using larger patient groups are needed to confirm the obtained results. No other associations were observed between the HCMV DNAemia and the examined *TLR* SNPs (*P* > 0.05).

### Haplotype analysis

Multiple-SNP analysis showed that the most common haplotype for the *TLR3* SNPs rs3775290, rs3775291, rs3775296 and the *TLR7* SNPs rs179008 and rs5741880 was CCCAT (21.5% for uninfected cases) and CTCAG (20.6% for children with HCMV infection). The CTCAG haplotype was associated with increased the risk of HCMV infection in an unadjusted model (OR 51.28, 95% CI 8.71–301.89), and this association reached statistical significance after Bonferroni’s correction (*P*^*c*^ < 0.001). The CTC and TTA haplotype of *TLR3* gene were associated with at least 20-fold increased the risk of HCMV infection in children (*P* = 0.0001 and *P* = 0.0071, respectively, see Table 6). The CTC haplotype association reached statistical significance after Bonferroni’s correction (*P*^*c*^ ≤ 0.0063). Performance of haplotype-trait association tests indicated that the TCAT haplotype of *TLR9* gene (rs5743836, rs187084, rs352139, rs352140) was also associated with HCMV infection, although this association did not reach statistical significance after Bonferroni’s correction (OR 4.23, 95% CI 1.30–13.70, *P* = 0.018; *P*^*c*^ = 0.005 significance level after Bonferroni’s correction). *TLR2* and *TLR7* haplotypes were not associated with the risk of infection.

**Table 6:** Haplotype analysis of *TLR3* SNPs in children with and without HCMV infection.

Haplotype			Frequency (%)		OR (95% CI)	*P*^*c*^
rs3775290	rs3775291	rs3775296	HCMV-infected	Uninfected		
C	C	C	16.61	50.95	1.00	NA
T	C	C	13.47	21.32	1.94 (0.65–5.76)	0.24
C	T	C	37.37	4.73	26.31 (5.30–130.68)	**0.0001**
C	T	A	4.28	10.86	1.61 (0.17–15.69)	0.68
T	T	A	1.22	4.36	20.91 (2.37–184.59)	0.0071
T	T	C	7.13	5.69	2.78 (0.57–13.52)	0.21
C	C	A	7.85	1.41	11.86 (0.91–154.56)	0.061
T	C	A	12.07	0.68	2.42 (0.10–57.63)	0.58

OR: odds ratio; 95% CI: 95% confidence interval; *P*, logistic regression model; NA: not available; *P*^*c*^, The significance level after Bonferroni’s correction for multiple testing is 0.0063 (raw *P*-value/8).

We found an association between the SNPs rs3775291 and rs3775296 of *TLR3* (*P* < 0.001) and *TLR7* SNPs rs179008 and rs5741880 (*P* < 0.001). In addition, the rs187084 was associated with rs352139 of *TLR9* gene (*P* = 0.002), that confirms previously described finding [24]. Linkage disequilibrium analysis revealed that the *TLR3* SNPs rs3775291 and rs3775296 exhibited a moderate level of disequilibrium (correlation coefficient r^2^ = 0.514, standardized disequilibrium coefficient D’ = 75). In contrast, all the other regions of genes were not in LD with each other (r^2^ < 0.2).

## Discussion

To our knowledge, no studies have been reported thus far exploring the possible involvement of *TLR3* and *TLR7* polymorphisms in the risk of HCMV infection in children. We selected the L412F SNP of the *TLR3* gene, which seems to be associated with HCMV infection and might have a direct functional correlation with virus replication. This *TLR3* gene variant appeared to be relatively common in HCMV-infected compared to uninfected pediatric patients. Moreover, the presence of the missense C>T mutation among subjects who were either heterozygous (CT) or homozygous for the minor allele (TT) was associated with a significant increase in symptomatic HCMV infection and viremia load. Hence, we suppose that the L412F variant of the *TLR3* gene may be one of the risk factor for HCMV disease in children.

Different studies strongly suggest that variations in genes encoding *TLRs* influence innate immune response to pathogens and disease outcome. In recent years, we have examined the relationship between single nucleotide polymorphisms in *TLRs* genes and HCMV infection in pediatric patients. At first, genetic polymorphisms in extracellular TLR2, that recognize the HCMV envelope gB and gH, and TLR4 were studied. The results showed that *TLR2* SNPs rs121917874, rs5743708, and *TLR4* SNP rs4986790 are not related to risk of HCMV infection in infants [28]. If endosomal TLR9 recognizes CpG oligonucleotides in the intracellular space, we decided to explore the correlation between *TLR9* polymorphisms and HCMV infection. We found that -1486T/C and 2848C/T SNPs were prevalent in HCMV-infected children and were associated with increased the risk of cytomegaly [24]. Encouraged by the results concerning the role of Toll-like receptors 3 and 7 in other herpesvirus infections [35–38], we recently decided to investigate whether common polymorphisms in these genes are associated with HCMV infection. The use of genotyping results for all *TLRs* SNPs in the current multiple analyses revealed that genetic variability in both *TLR3* and *TLR9* genes may have consequences that can contribute to HCMV disease.

The ability of TLR3 to sense dsRNA and stimulate the production of type I IFNs and inflammatory cytokines suggests that this receptor plays an important role in antiviral defense. This receptor is localized mainly on intracellular vesicles with some cell surface expression. After binding the dsRNA motif on pathogens, TLR3 dimerizes and recruits the TIR domain-containing adaptor protein-inducing IFNβ (TRIF) to the endosome. TRIF recruitment results in signaling to activate the transcription factors IFN regulatory factor 3 (IRF3) and nuclear factor-κB (NF-ĸB), which induce type I IFN expression [8, 39]. Moreover, stimulation of TLR3 with poly(I:C) induced expression of IFN-β and IFN-inducible genes, including pro-inflammatory cytokines and chemokines [39]. The relationship between TLR3 and viral infection is highly complex and virus-specific. The role of TLR3 in HCMV infection is still unclear, while its function in mediating immunity against MCMV infection was demonstrated [40]. As TLR3-deficient mice display inadequate responses to MCMV infection, it is suggested that TLR3 plays an important role in cytomegalovirus recognition [40]. Mice with a mutation in TRIF were found to be more susceptible to MCMV infection [41]. In contrast, no role for TLR3 signaling could be demonstrated during experiments in which TLR3 was silenced by siRNA before infection with a wild-type isolate of HCMV. The early HCMV-triggered immune response of human monocyte-derived dendritic cells appears to be independent of the TLR3 pathway [42]. In addition, Edelmann et al. [43] demonstrated that TLR3 is not required for the generation of effective antiviral responses because the absence of TLR3 does not either alter viral pathogenesis or impair the host's generation of adaptive antiviral responses to different viruses, including MCMV.

Increasing evidence suggests that the mutation that leads to the replacement of the leucine (L) residue in amino acid position 412 by a phenylalanine (F) residue (L412F) in the *TLR3* ectodomain plays an important role in viral infection. Our data revealed that the L412F minor allele frequency was 50% in the young children with HCMV infection in comparison to 25.6% in the uninfected Polish children. This is significantly higher than the allele frequency of the European (32%) and the overall populations (23%) (Table 2). The heterozygous or homozygous recessive genotypes of this SNP were consistently associated with a higher risk of HCMV infection and disease development. An additional multiple-SNP analysis, including eleven *TLR2*, *TLR3*, *TLR7*, and *TLR9* polymorphisms, confirmed the essential role of the *TLR3* SNP rs3775291 in cytomegaly. Moreover, these genotypes were detected in patients with a high viremia level. We suggest that the L412F mutation within the *TLR3* gene results in a signaling impairment and could be associated with HCMV infection. In experiments *in vitro*, two amino acid changes (L412F and N284I) in the exon-coding region were demonstrated to impair TLR3 signaling in response to its ligand [44]. It was previously shown that genetic variation in *TLR3* affects the host’s susceptibility to other herpesvirus infections [35, 45]. The common L412F and the rare non-synonymous substitution rs121434431 (P554S) SNPs were associated with HSV-1 encephalitis [36]. The 412F allele carriers were also associated with HSV-2 meningitis [37] and recurrent herpes labialis [38]. The SNPs at rs3775291 and rs13126816 in the *TLR3* gene were correlated with a reduced incidence of genital HSV-2 infection but did not affect disease severity in the infected hosts [35]. The L412F SNP was not associated with HCMV infection in renal transplant recipients, although it was linked with the incidence of acute rejection and graft function [21]. This polymorphism was also over-represented in other diseases, including liver recipients infected with human hepatitis C virus [46], infants with bronchiolitis [47], and individuals with asthma [40] or Japanese encephalitis [48]. The L412F SNP was also linked with resistance against HIV infection in the Italian population [49]. Lower HIV-1 replication and more vigorous immunological responses to TLR3 stimulation were observed in cells from individuals with an L412F mutation in at least one allele when compared with cells from dominant homozygotes.

The structure of the human TLR3 ectodomain revealed a horseshoe shape, characteristic of proteins containing multiple leucine-rich repeats [50]. The conserved L412 residue exists within the TLR3 ectodomain, and L412F mutation is very likely to be disruptive to the structure and function of TLR3 [40]. It may alter the hydrophobic interactions and affect the glycosylation of neighboring residues that are critical for receptor function and proper TLR3 signaling [26, 40]. Because the rs3775291 SNP is not in the promoter region, it should not influence the mRNA and protein expression of TLR3. However, it was found that this SNP reduces the cell-surface, but not intracellular, TLR3 expression [40]. Moreover, the binding capacity of the L412F mutant protein for dsRNA was reduced to half of the wild-type level [51]. As the N413A mutation significantly reduces TLR3 signaling [52], it is possible that L412F could either affect the glycosylation of asparagine 413 or hinder the interaction of its glycan moiety with dsRNA [53]. This may explain the reduced signaling activity of *TLR3* -412F. Nahum et al. [26] have found that patient’s cells carrying the L412F variant have reduced IFNγ and TNFα in response to stimulation with cytomegalovirus than did cells with the wild-type genotype. This may explain the clinical manifestations exhibited by patients carrying this receptor, such as recurrent viral infections and severe manifestations related to HCMV infection.

We did not find an association between *TLR7* SNP genotypes and the risk of HCMV infection, although higher viremia levels were detected in carriers of the heterozygous genotype of the *TLR7* intronic SNP rs5741880. The polymorphisms in exon 3 of the X-linked *TLR7* gene have been suggested to play a role in the immune response to HCMV glycoproteins. Surprisingly, it was found that SNP rs179008, associated with an amino acid change from glutamine to leucine in position 11 (G11L), was related with variability in HCMV gB antibodies [27]. Women with the homozygous variant of this SNP demonstrated a higher vaccination-induced antibody response to gB than did heterozygotes or homozygotes for this allele. Thus far, a functional effect of this SNP was confirmed in other viral diseases, e.g., chronic HCV infection [54]. The findings of experiments *in vitro* with plasmacytoid dendritic cells (PDCs) suggest that HCMV induces IFNα secretion from these cells through the engagement of the TLR7 and/or TLR9 pathways [55]. Moreover, Epstein-Barr virus interacts with the TLR7 pathway and enhances B cell proliferation [56]. Although TLR7 alone does not appear to have a strong role in cytomegalovirus recognition, TLR7/TLR9 and TLR3/TLR7/TLR9 combined deficiencies were shown to impair responses to MCMV [57, 58].

This study has a number of strengths and limitations. The main strength of the present study is the clinical evaluation of children with HCMV infection and the genetic homogeneity of the study population. We selected potentially functional SNPs for our study to increase our chances of identifying polymorphisms related to HCMV disease. However, no population frequencies data were available for these SNPs, so we used HCMV-seronegative newborn infants from our previous cohort study. The genetic variability of our cohort and the European population is very similar. Since HCMV infection was diagnosed in the children after the three weeks of age, it is possible that the patients represent two different populations: congenital and postnatal HCMV infection. Although the number of cases remains small for genetic studies we believe that we have found *TLR* polymorphisms associated with primary HCMV infection. Significant differences in genotype distribution in case-control association study indicate that these polymorphisms remain an important risk factor. Further studies including large numbers of patients are necessary to confirm our findings. Functional analyses may contribute to the understanding of the role of the studied genes in HCMV infection.

In conclusion, the results demonstrate that the L412F polymorphism in the *TLR3* gene is associated with HCMV disease in children. We suggest that this polymorphism conferred reduced secretion of cytokines with antiviral activity, such as IFN and TNFα secretion, and consequently, it could be a genetic risk factor for the development of cytomegaly. Because the L412F SNP is encoded in the exon region, we consider it important to extend this study in the future by performing expression and functional analysis of the TLR3 protein with this SNP. Better understanding of how the host recognizes HCMV and mediates cellular signaling and immune responses is crucial for improving prophylaxis and therapeutic approaches against this important viral pathogen.

## References

[1] Griffiths P, Baraniak I, Reeves M. The pathogenesis of human cytomegalovirus. J Pathol. 2015;235: 288–297. 10.1002/path.4437 25205255

[2] Kenneson A, Cannon MJ. Review and meta-analysis of the epidemiology of congenital cytomegalovirus (CMV) infection. Rev Med Virol. 2007;179(4): 253–276. 10.1002/rmv.535 17579921

[3] Wang C, Zhang X, Bialek S, Cannon MJ. Attribution of congenital cytomegalovirus infection to primary versus non-primary maternal infection. Clin Infect Dis. 2011;52(2): e11–e13. 10.1093/cid/ciq085 21288834

[4] Jensen S, Thomsen AR. Sensing of RNA viruses: a review of innate immune receptors involved in recognizing RNA virus invasion. J Virol. 2012;86: 2900–2910. 10.1128/JVI.05738-11 22258243 PMC3302314

[5] Kang D C, Gopalkrishnan RV, Wu Q, Jankowsky E, Pyle AM, Fisher PB. mda-5: An interferon-inducible putative RNA helicase with double-stranded RNA-dependent ATPase activity and melanoma growth-suppressive properties. Proc Natl Acad Sci USA. 2002;99: 637–642. 10.1073/pnas.022637199 11805321 PMC117358

[6] Kato H, Takeuchi O, Sato S, Yoneyama M, Yamamoto M, Matsui K, et al Differential roles of MDA5 and RIG-I helicases in the recognition of RNA viruses. Nature. 2006;441: 101–105. 10.1038/nature04734 16625202

[7] Yoneyama M, Kikuchi M, Natsukawa T, Shinobu N, Imaizumi T, Miyagishi M, et al The RNA helicase RIG-I has an essential function in double-stranded RNA-induced innate antiviral responses. Nat Immunol. 2004;5: 730–737. 10.1038/ni1087 15208624

[8] Alexopoulou L, Holt AC, Medzhitov R, Flavell RA. Recognition of double-stranded RNA and activation of NF-kappaB by Toll-like receptor 3. Nature 2001;413: 732–738. 10.1038/35099560 11607032

[9] Guillot L, Le Goffic R, Bloch S, Escriou N, Akira S, Chignard M, et al Involvement of toll-like receptor 3 in the immune response of lung epithelial cells to double-stranded RNA and influenza A virus. J Biol Chem. 2005;280: 5571–5580. 10.1074/jbc.M410592200 15579900

[10] Hardarson HS, Baker JS, Yang Z, Purevjav E, Huang CH, Alexopoulou L, et al Toll-like receptor 3 is an essential component of the innate stress response in virus-induced cardiac injury. Am J Physiol Heart Circ Physiol. 2007;292(1): H251–H258. 10.1152/ajpheart.00398.2006 16936008

[11] Kumar H, Kawai T, Akira S. Toll-like receptors and innate immunity. Biochem Biophys Res Commun. 2009;388(4): 621–625. 10.1016/j.bbrc.2009.08.062 19686699

[12] Diebold SS, Kaisho T, Hemmi H, Akira S, Reis e Sousa C. Innate antiviral responses by means of TLR7-mediated recognition of single-stranded RNA. Science. 2004;303(5663): 1529–1531. 10.1126/science.1093616 14976261

[13] Lund JM, Alexopoulou L, Sato A, Karow M, Adams NC, Gale NW, et al Recognition of single-stranded RNA viruses by Toll-like receptor 7. Proc Natl Acad Sci USA. 2004;101: 5598–5603. 10.1073/pnas.0400937101 15034168 PMC397437

[14] Krug A, Luker GD, Barchet W, Leib DA, Akira S, Colonna M. Herpes simplex virus type 1 activates murine natural interferon-producing cells through toll-like receptor 9. Blood. 2004;103(4): 1433–1437. 10.1182/blood-2003-08-2674 14563635

[15] Lund J, Sato A, Akira S, Medzhitov R, Iwasaki A. Toll-like receptor 9-mediated recognition of Herpes simplex virus-2 by plasmacytoid dendritic cells. J Exp Med. 2003;198(3): 513–520. 10.1084/jem.20030162 12900525 PMC2194085

[16] Kijpittayarit S, Eid AJ, Brown RA, Paya CV, Razonable RR. Relationship between Toll-like receptor 2 polymorphism and cytomegalovirus disease after liver transplantation. Clin Infect Dis. 2007;44: 1315–1320. 10.1086/514339 17443468

[17] Kang SH, Abdel-Massih RC, Brown RA, Dierkhising RA, Kremers WK, Razonable RR. Homozygosity for the toll-like receptor 2 R753Q single-nucleotide polymorphism is a risk factor for cytomegalovirus disease after liver transplantations. J Infect Dis. 2012;205: 639–646. 10.1093/infdis/jir819 22219347 PMC3266129

[18] Cervera C, Lozano F, Saval N, Gimferrer I, Ibañez A, Suárez B, et al The influence of innate immunity gene receptors polymorphisms in renal transplant infections. Transplantation. 2007;83: 1493–1500. 10.1097/01.tp.0000264999.71318.2b 17565323

[19] Carvalho A, Cunha C, Carotti A, Aloisi T, Guarrera O, Di Ianni M, et al Polymorphisms in Toll-like receptor genes and susceptibility to infections in allogeneic stem cell transplantation. Exp Hematol. 2009;37: 1022–1029. 10.1016/j.exphem.2009.06.004 19539691

[20] Xiao HW, Luo Y, Lai XY, Shi JM, Tan YM, He JS, et al Donor TLR9 gene tagSNPs influence susceptibility to a GVHD and CMV reactivation in the allo-HSCT setting without polymorphisms in the TLR4 and NOD2 genes. Bone Marrow Transplant. 2014;49: 241–247. 10.1038/bmt.2013.160 24121213

[21] Krüger B, Banas MC, Walberer A, Böger CA, Farkas S, Hoffmann U, et al A comprehensive genotype-phenotype interaction of different Toll-like receptor variations in a renal transplant cohort. Clin Sci (Lond). 2010;119: 535–544. 20604744 10.1042/CS20100190

[22] Seo S, Fan W, Hansen JA, Storer BE, Pergam SA, Green ML, et al Evaluation of published single-nucleotide polymorphisms (SNPs) associated with cytomegalovirus infection and disease after hematopoietic cell transplantation in a large Caucasian cohort. Blood. 2014;124: 182. conference paper

[23] Wujcicka W, Paradowska E, Studzińska M, Gaj Z, Wilczyński J, Leśnikowski Z, et al TLR9 2848 GA heterozygotic status possibly predisposes fetuses and newborns to congenital infection with human cytomegalovirus. PLoS One. 2015;10: e0122831 10.1371/journal.pone.0122831 25844529 PMC4386761

[24] Paradowska E, Jabłońska A, Studzińska M, Skowrońska K, Suski P, Wiśniewska-Ligier M, et al TLR9 -1486T/C and 2848C/T SNPs Are Associated with Human Cytomegalovirus Infection in Infants. PLoS One. 2016;11(4): e0154100 10.1371/journal.pone.0154100 27105145 PMC4841553

[25] Taniguchi R, Koyano S, Suzutani T, Goishi K, Ito Y, Morioka I, et al Polymorphisms in TLR-2 are associated with congenital cytomegalovirus (CMV) infection but not with congenital CMV disease. Int J Infect Dis. 2013;17(12): e1092–7. 10.1016/j.ijid.2013.06.004 23906542

[26] Nahum A, Dadi H, Bates A, Roifman CM. The biological significance of TLR3 variant, L412F, in conferring susceptibility to cutaneous candidiasis, CMV and autoimmunity. Autoimmun Rev. 2012;11(5): 341–347. 10.1016/j.autrev.2011.10.007 22024499

[27] Arav-Boger R, Wojcik GL, Duggal P, Ingersoll RG, Beaty T, Pass RF, et al Polymorphisms in Toll-like receptor genes influence antibody responses to cytomegalovirus glycoprotein B vaccine. BMC Res Notes. 2012;5: 140 10.1186/1756-0500-5-140 22414065 PMC3317442

[28] Jabłońska A, Paradowska E, Studzińska M, Suski P, Nowakowska D, Wiśniewska-Ligier M, et al Relationship between toll-like receptor 2 Arg677Trp and Arg753Gln and toll-like receptor 4 Asp299Gly polymorphisms and cytomegalovirus infection. Int J Infect Dis. 2014;25: 11–15. 10.1016/j.ijid.2014.04.001 24813591

[29] Noguchi E, Nishimura F, Fukai H, Kim J, Ichikawa K, Shibasaki M, et al An association study of asthma and total serum immunoglobin E levels for Toll-like receptor polymorphisms in a Japanese population. Clin Exp Allergy. 2004;34(2): 177–183. 14987294 10.1111/j.1365-2222.2004.01839.x

[30] Cheng PL, Eng HL, Chou MH, You HL, Lin TM. Genetic polymorphisms of viral infection-associated Toll-like receptors in Chinese population. Transl Res. 2007;150(5): 311–318. 10.1016/j.trsl.2007.03.010 17964520

[31] Arslan S, Engin A, Özbilüm N, Bakır M. Toll-like receptor 7 Gln11Leu, c.4-151A/G, and +1817G/T polymorphisms in Crimean Congo hemorrhagic fever. J Med Virol. 2015;87(7): 1090–1095. 10.1002/jmv.24174 25879168

[32] Paradowska E, Przepiórkiewicz M, Nowakowska D, Studzińska M, Wilczyński J, Emery VC, et al Detection of cytomegalovirus in human placental cells by polymerase chain reaction. APMIS. 2006;114: 764–771. 10.1111/j.1600-0463.2006.apm_31.x 17078856

[33] Sole X, Guino E, Valls J, Iniesta R, Moreno V. SNPStats: a web tool for the analysis of association studies. Bioinformatics. 2006;22(15): 1928–1929. 10.1093/bioinformatics/btl268 16720584

[34] Barrett JC, Fry B, Maller J, Daly MJ. Haploview: analysis and visualization of LD and haplotype maps. Bioinformatics. 2005;21(2): 263–265. 10.1093/bioinformatics/bth457 15297300

[35] Svensson A, Tunbäck P, Nordström I, Padyukov L, Eriksson K. Polymorphisms in Toll-like receptor 3 confer natural resistance to human herpes simplex virus type 2 infection. J Gen Virol. 2012;93: 1717–1724. 10.1099/vir.0.042572-0 22552940

[36] Zhang SY, Jouanguy E, Ugolini S, Smahi A, Elain G, Romero P, et al TLR3 deficiency in patients with herpes simplex encephalitis. Science. 2007;317(5844): 1522–1527. 10.1126/science.1139522 17872438

[37] Willmann O, Ahmad-Nejad P, Neumaier M, Hennerici MG, Fatar M. Toll-like receptor 3 immune deficiency may be causative for HSV-2-associated mollaret meningitis. Eur Neurol. 2010;63(4): 249–251. 10.1159/000287585 20375513

[38] Yang CA, Raftery MJ, Hamann L, Guerreiro M, Grütz G, Haase D, et al Association of TLR3-hyporesponsiveness and functional TLR3 L412F polymorphism with recurrent herpes labialis. Hum Immunol. 2012;73(8): 844–851. 10.1016/j.humimm.2012.04.008 22537752

[39] Yamamoto M, Sato S, Hemmi H, Hoshino K, Kaisho T, Sanjo H, et al Role of adaptor TRIF in the MyD88-independent toll-like receptor signaling pathway. Science. 2003;301(5633): 640–643. 10.1126/science.1087262 12855817

[40] Tabeta K, Georgel P, Janssen E, Du X, Hoebe K, Crozat K, et al Toll-like receptors 9 and 3 as essential components of innate immune defense against mouse cytomegalovirus infection. Proc Natl Acad Sci USA. 2004;101(10): 3516–3521. 10.1073/pnas.0400525101 14993594 PMC373494

[41] Hoebe K, Du X, Georgel P, Janssen E, Tabeta K, Kim SO, et al Identification of Lps2 as a key transducer of MyD88-independent TIR signalling. Nature. 2003;424: 743–748. 10.1038/nature01889 12872135

[42] Mezger M, Bonin M, Kessler T, Gebhardt F, Einsele H, Loeffler J. Toll-like receptor 3 has no critical role during early immune response of human monocyte-derived dendritic cells after infection with the human cytomegalovirus strain TB40E. Viral Immunol. 2009;22(6): 343–351. 10.1089/vim.2009.0011 19951172

[43] Edelmann KH, Richardson-Burns S, Alexopoulou L, Tyler KL, Flavell RA, Oldstone MB. Does Toll-like receptor 3 play a biological role in virus infections? Virology. 2004;322: 231–238. 10.1016/j.virol.2004.01.033 15110521

[44] Ranjith-Kumar CT, Miller W, Sun J, Xiong J, Santos J, Yarbrough I, et al Effects of single nucleotide polymorphisms on toll-like receptor 3 activity and expression in cultured cells. J Biol Chem. 2007;282(24): 17696–17705. 10.1074/jbc.M700209200 17434873

[45] Netea MG, Wijmenga C, O'Neill LA. Genetic variation in Toll-like receptors and disease susceptibility. Nat Immunol. 2012;13(6): 535–542. 10.1038/ni.2284 22610250

[46] Lee SO, Brown RA, Razonable RR. Association between a functional polymorphism in Toll-like receptor 3 and chronic hepatitis C in liver transplant recipients. Transpl Infect Dis. 2013;15(2): 111–119. 10.1111/tid.12033 23240626

[47] Nuolivirta K, He Q, Vuononvirta J, Koponen P, Helminen M, Korppi M. Toll-like receptor 3 L412F polymorphisms in infants with bronchiolitis and postbronchiolitis wheezing. Pediatr Infect Dis J. 2012;31(9): 920–923. 10.1097/INF.0b013e31825aff25 22549436

[48] Biyani S, Garg RK, Jain A, Malhotra HS, Kumar R, Prakash S, et al Toll-like receptor-3 gene polymorphism in patients with Japanese encephalitis. J Neuroimmunol. 2015;286: 71–76. 10.1016/j.jneuroim.2015.07.010 26298326

[49] Sironi M, Biasin M, Cagliani R, Forni D, De Luca M, Saulle I, et al A common polymorphism in TLR3 confers natural resistance to HIV-1 infection. J Immunol. 2012;188(2): 818–823. 10.4049/jimmunol.1102179 22174453

[50] Bell JK, Botos I, Hall PR, Askins J, Shiloach J, Davies DR, et al The molecular structure of the TLR3 extracellular domain. J Endotoxin Res. 2006;12(6): 375–378. 10.1179/096805106X118780 17254392

[51] Zhou P, Fan L, Yu KD, Zhao MW, Li XX. Toll-like receptor 3 C1234T may protect against geographic atrophy through decreased dsRNA binding capacity. FASEB J. 2011;25: 3489–3495. 10.1096/fj.11-189258 21712495

[52] Sun J, Duffy KE, Ranjith-Kumar CT, Xiong J, Lamb RJ, Santos J, et al Structural and functional analyses of the human Toll-like receptor 3. Role of glycosylation. J Biol Chem 2006;281(16): 11144–11151. 10.1074/jbc.M510442200 16533755

[53] Gorbea C, Makar KA, Pauschinger M, Pratt G, Bersola JL, Varela J, et al A role for Toll-like receptor 3 variants in host susceptibility to enteroviral myocarditis and dilated cardiomyopathy. J Biol Chem. 2010;285: 23208–23223. 10.1074/jbc.M109.047464 20472559 PMC2906314

[54] Askar E, Ramadori G, Mihm S. Toll-like receptor 7 rs179008/Gln11Leu gene variants in chronic hepatitis C virus infection. J Med Virol. 2010;82(11): 1859–1868. 10.1002/jmv.21893 20872712

[55] Varani S, Cederarv M, Feld S, Tammik C, Frascaroli G, Landini MP, et al Human cytomegalovirus differentially controls B cell and T cell responses through effects on plasmacytoid dendritic cells. J Immunol. 2007;179(11): 7767–7776. 18025223 10.4049/jimmunol.179.11.7767

[56] Martin HJ, Lee JM, Walls D, Hayward SD. Manipulation of the toll-like receptor 7 signaling pathway by Epstein-Barr virus. J Virol. 2007;81(18): 9748–9758. 10.1128/JVI.01122-07 17609264 PMC2045431

[57] Zucchini N, Bessou G, Traub S, Robbins SH, Uematsu S, Akira S, et al Cutting edge: Overlapping functions of TLR7 and TLR9 for innate defense against a herpesvirus infection. J Immunol. 2008;180(9): 5799–5803. 18424698 10.4049/jimmunol.180.9.5799

[58] Crane MJ, Gaddi PJ, Salazar-Mather TP. UNC93B1 Mediates Innate Inflammation and Antiviral Defense in the Liver during Acute Murine Cytomegalovirus Infection. PLoS10.1371/journal.pone.0039161 PMC3377622 22723955

